# Impact of the Polymorphism *rs9264942* near the *HLA-C* Gene on HIV-1 DNA Reservoirs in Asymptomatic Chronically Infected Patients Initiating Antiviral Therapy

**DOI:** 10.1155/2017/8689313

**Published:** 2017-12-28

**Authors:** Laura Herráiz-Nicuesa, Diana Carolina Hernández-Flórez, Lara Valor, Sonia García-Consuegra, Juan Paulo Navarro-Valdivieso, Eduardo Fernández-Cruz, Carmen Rodríguez-Sainz

**Affiliations:** ^1^Servicio de Inmunología Clínica, Hospital General Universitario Gregorio Marañón, Madrid, Spain; ^2^Instituto de Investigación Sanitaria Gregorio Marañón, Madrid, Spain

## Abstract

Several genome-wide association studies have identified a polymorphism located 35 kb upstream of the coding region of *HLA-C* gene (*rs9264942*; termed −35 C/T) as a host factor significantly associated with the control of HIV-1 viremia in untreated patients. The potential association of this host genetic polymorphism with the viral reservoirs has never been investigated, nor the association with the viral control in response to the treatment. In this study, we assess the influence of the polymorphism −35 C/T on the outcome of virus burden in 183 antiretroviral-naïve HIV-1-infected individuals who initiated antiviral treatment (study STIR-2102), analyzing HIV-1 RNA viremia and HIV-1 DNA reservoirs. The *rs9264942* genotyping was investigated retrospectively, and plasma levels of HIV-1 RNA and peripheral blood mononuclear cell- (PBMC-) associated HIV-1 DNA were compared between carriers and noncarriers of the protective allele −35 C before antiretroviral therapy (ART), one month after ART and at the end of the study (36 months). HIV-1 RNA and HIV-1 DNA levels were both variables significantly different between carriers and noncarriers of the allele −35 C before ART. HIV-1 DNA levels remained also significantly different one month posttherapy. However, this protective effect of the −35 C allele was not maintained after long-term ART.

## 1. Introduction

The clinical outcome of HIV-1 infection is highly variable and determined by complex interactions between virus, host, and environment. Part of this epidemiological heterogeneity could be attributed to host genetic factors, which have been extensively studied using whole genome approaches (reviewed in [1, 2]). Several genome-wide association studies (GWAS) have identified a polymorphism located 35 kb upstream of the coding region of the gene *HLA-C* (−35 C/T; *rs9264942*) as a host factor significantly associated with the control of HIV-1 viremia. Fellay et al. reported the first AIDS GWAS in 2007 [3]. Their study tracked the viral set point (mean plasma RNA level over several months once the immune system has settled to a steady-state level after primo-infection) in 486 European AIDS patients (Cohort EURO-CHAVI) that were genotyped. The variant *rs9264942* was the second most significant independent hit in the EURO-CHAVI study and resulted the top hit in the International HIV Controller Study involving 974 controllers, as reported three years later [4]. Another HIV-1 viral set point GWAS with 2554 Caucasian participants provided overwhelming confirmation of the SNP *rs9264942* [5]. This SNP also associates strongly with differences in *HLA-C* expression levels. The protective allele (−*35 C*) leads to a lower viral load and is associated with higher expression of the *HLA-C* gene [3].

Recently, more work has focused on elucidating the functional significance of the *−35 C/T* SNP, and several groups now have demonstrated HLA-C surface expression to be a key element in the control of HIV viral load [6]. HLA-C surface expression has been correlated with the presence of microRNA binding sites that affect *HLA-C* expression and control of HIV disease. It has been identified a binding site for the microinterference RNA, miR-148a, that is present in the sequence of some *HLA-C* alleles, but missing in others due to polymorphism in the region. This polymorphism includes an insertion/deletion variant at the position 263 of the 3′UTR of the *HLA-C* gene, which is in very strong linkage disequilibrium with *rs9264942* in Caucasians [7]. The linkage disequilibrium of the −35 SNP with the 3′UTR miRNA-148a binding site polymorphisms offers a functional explanation for the observed differences in HLA-C surface expression among individuals and the associated control of HIV disease. Globally, all these data support a role for *HLA-C* in early and/or chronic infection [8, 9].

*HLA-C* is the most recently evolved of the classical *MHC-I* alleles and is restricted to humans and great apes [10, 11]; and fewer alleles of *HLA-C* have been identified. HLA-C plays a dual role in that it can present antigens to CTLs and it can inhibit natural killer (NK) cell (and possibly also CTL) lysis via its interaction with inhibitory receptors (killer immunoglobulin-like receptors, KIR). For reasons that are poorly understood, HLA-C is normally expressed on the cell surface at levels approximately 10-fold less than most *HLA-A* and *HLA-B* allotypes [12–14]. In HIV-infected individuals, HLA-A and HLA-B molecules expressed on the surface of the infected cells are preferentially downmodulated by the viral accessory protein Nef, but not HLA-C molecules [15], and so HLA-C may have a unique role in presenting antigens to CTLs in HIV disease and modulating cytotoxicity.

Since the first GWAS study of Fellay, involving the variant *rs9264942* near the HLA-C as a genetic determinant in HIV-1 infection, most of the GWAS performed to date in cohorts involving various outcomes of HIV infection are focus on the viremia or the control of circulating virus and all of them have been designed in the absence of antiviral therapy. Viral burden has been extensively investigated in the HLA-C background analyzing plasmatic HIV-1 RNA, but HIV-1 reservoirs, particularly HIV-1 DNA levels are lesser known. The potential association of the variant *rs9264942* with the viral control under antiviral treatment is also unknown.

The objective of this study was to assess the influence of the polymorphism *rs9264942* on the outcome of virus burden in 183 antiretroviral-naïve individuals who initiated antiviral treatment (study STIR-2102), analyzing HIV-1 RNA viremia and HIV-1 DNA reservoirs associated to PBMC. We quantified HIV-1 RNA from plasma and HIV-1 DNA obtained from PBMCs before antiretroviral therapy (ART) and during the treatment with a follow-up of 36 months.

## 2. Materials and Methods

### 2.1. Patients

One hundred eighty-three patients with asymptomatic HIV-1 chronic infection who had participated in the clinical trial STIR-2102 [16, 17] were retrospectively genotyped for the polymorphism *rs9264942*. The study STIR-2102 was a multicenter, randomized, double-blinded, placebo-controlled phase II clinical trial of antiretroviral therapy (ART) in combination with an HIV-1 immunogen (Remune®) in antiretroviral-naïve HIV-1-infected subjects with CD4^+^ T lymphocytes between 300 and 700 cells/*μ*L. ART consisted of zidovudine and didanosine. Patients started therapy one month prior randomization to receive ART plus the immunogen (*N* = 86) or ART plus placebo (*N* = 97). Treatment arms showed no differences in baseline factors, such as age, gender, risk group, viral load, and CD4^+^ T cells. The baseline characteristics for the study STIR-2102 are shown in Table 1. The immunovorological response to the therapy was similar at the end of the study with no significant differences between both arms. Prior to the commencement of the study STIR-2102, conducted between 1997 and 2001, Institutional Review Board approval by each participant hospital was obtained. Furthermore, informed consents from all participants, jointly with the review and approval of the protocol by the Spanish Agency of Medicament and Sanitary Products, were also obtained prior to the initiation of the trial.

### 2.2. Plasma HIV-1 RNA Viremia and CD4^+^ T Cell Subset

Laboratory test including CD4^+^ T cell counts and plasma HIV-1 RNA levels were carried out every three months throughout the study (36 months). Plasma levels of HIV-1 RNA were assessed using the Amplicor assay (Hoffman La Roche, Nutley, NJ) with a lower limit of quantification of 200 copies/mL (2.30 log_10_ copies/mL) [16, 17].

### 2.3. Polymorphism rs9264942 Genotyping Assessment

Blood samples were collected from patients during the trial, and DNA was isolated from the peripheral blood mononuclear cells by standard protocols. PBMC were separated by Ficoll gradient (Pharmacia, Uppsala, Sweden) and stored as dry pellets at −80°C. DNA was purified from each PBMC pellet by Wizard Genomic DNA Purification Kit (Promega Corporation, Madison, WI, USA), and DNA content was determined by spectrophotometric analysis.

The *rs9264942* genotyping was retrospectively performed by real-time PCR amplification with the LightSNiP *rs9264942 HLA-C* (TIB MOLBIOL GmbH, Berlin, Germany; under license from Roche Diagnostics GmbH) using LightCycler® FastStart DNA Master HybProbe (Roche Diagnostics) and a LightCycler 1.5 Instrument, according to the manufacturer's protocol. The genotype was verified by sequencing.

### 2.4. HIV-1 DNA Level

HIV-1 DNA was quantified with a real-time quantitative PCR method using SYBR Green and primers of the *pol* gene as previously described [18]. Fluorescence was monitored specifically in a single point of each amplification cycle, allowing the determination of initial DNA copies through comparison with a standard curve constructed with DNA from the T-lymphoblastoid cell lines Jurkat and 8E5LAV that do not contain HIV-1 LAV or only in the amount of one single copy of HIV-1 LAV per cell, respectively. The detection limit of our assay was 250 copies/10^6^ PBMC. Results of the PBMC-associated HIV-1 DNA in this cohort of patients were reported partially in a previous study [18].

### 2.5. Endpoints and Statistical Analysis

In STIR-2102 trial, the virologic endpoint was defined as time to the first increase of viral load above 5000 copies per milliliter and the immunological endpoint as time to the first decrease of CD4^+^ T cell count below 250 cells/*μ*L [16]. Subjects who at the end of the study at month 36 had not developed a primary endpoint were censored at the last available visit. Kaplan-Meier analysis was used to construct event-free survival curves, which were compared using the log-rank test. Adjusted hazard ratios (HRs) and the 95% confidence intervals (CIs) were calculated using multivariate Cox proportional-hazard models. Proportional hazard assumptions were assessed as previously described [19]. Virologic data did not show a normal distribution and were expressed as median. Mann–Whitney test was used to compare groups. We used the Statistical Package for the Social Sciences (SPSS) and Stata for statistical analysis.

## 3. Results

### 3.1. Distribution of the rs9264942 Genotypes in the Study Cohort

Among the 183 chronically infected study subjects, thirty-four (18.6%) displayed the genotype homozygous for the protective allele (−*35 CC*), whereas ninety-five (51.9%) were heterozygous (−*35 CT*) and fifty-four were homozygous for the allele −*35* T (29.5%) (Table 2). The allelic frequency of the minor allele (−*35 C*) was 44.5%. The study population was in Hardy-Weinberg equilibrium.

### 3.2. Association of the Allele −35 C with HIV-1 Viral Load

To evaluate the impact of the protective allele *−35 C* on the steady-state plasma HIV-1 RNA levels in the study cohort, we compared viral load levels between carriers and noncarriers of the allele, at the baseline when individuals were ART-naïve. Patients included in this study were asymptomatic and maintained a relatively stable viral load off therapy. The analysis revealed decreased set points of the plasmatic viremia in *−35 C* carriers as compared to those with the genotype *−35 TT* reaching statistical significance (*p* = 0.035; median comparison for independent samples). HIV-1 DNA levels were also significantly different between patients with and without the allele *−35 C* (*p* < 0.001; median comparison for independent samples, Table 3). This allele was associated with lesser levels of HIV-1 DNA in PBMCs.

HIV-1 DNA levels in PBMCs remained also significantly different one month posttherapy [median = 425 cop/10^−6^ PBMCs (max = 18,855, min = 250 cop/10^−6^ PBMCs) in *−35 C* carriers versus median = 1080 cop/10^−6^ PBMCs (max = 10,645, min = 250 cop/10^−6^ PBMCs) in the individuals with the genotype −35 TT; *p* = 0.010, median comparison for independent samples].

### 3.3. Association of the Allele −35 C with HIV-1 Disease Progression in Response to the Therapy

**Figure 1:**
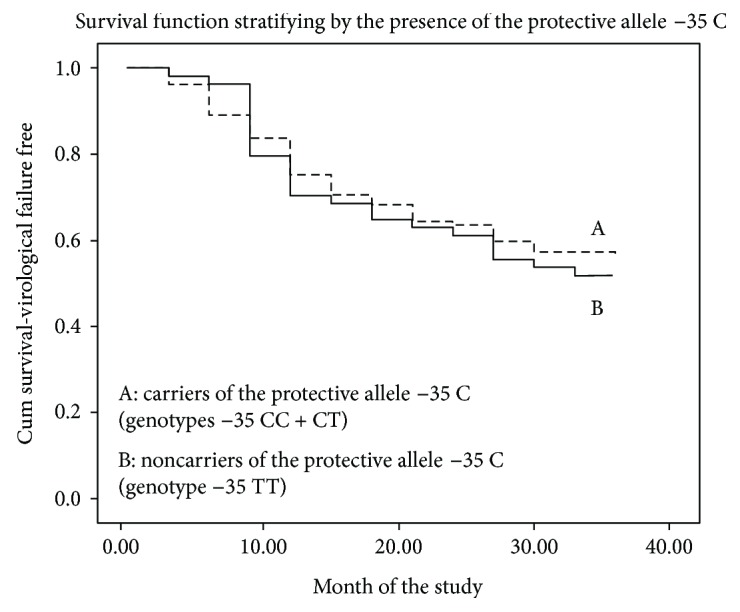
Kaplan-Meier estimates of virological failure-free survival during antiviral therapy according to the stratification by the presence of the protective allele −35 C (SNP *rs9264942*).

We did not find any protective effect of the allele *−35 C* in Kaplan-Meier survival analysis of the 183 therapy-naïve chronically infected individuals during the thirty-six months of the study STIR-2102 after the initiation of the antiretroviral therapy. Carriers and noncarriers of the allele −*35 C* reached the study endpoint (VL > 5000 copies/mL or CD4 T cells < 250 cells/mL) without showing differences statistically significant [mean time to the endpoint = 30.0 months (CI, 27.4–32.5) versus mean time to the endpoint = 29.0 months (CI, 25.1–32.9), respectively; log-rank test *p* = 0.616; Figure 1].

A multivariate Cox model analysis was performed by introducing as variable the presence of the protective allele *−35 C* and covariating with the variables HIV-1 RNA level and HIV-1 DNA level at the baseline pre-ART (Table 4). HIV-1 DNA and HIV-1 RNA were both independently associated with virological failure. The adjusted HR was significant for each 1-log_10_ increase in the baseline HIV-1 RNA level (adjusted HR, 1.86 [95% IC, 1.27–2.72, *p* = 0.001]) and for each 1-log_10_ increase in the baseline HIV-1 DNA (HR 1.74 [95% CI, 1.10–2.77, *p* = 0.018]). The protective allele *−35 C* was not significantly associated with the virological failure in this model (*p* = 0.69). Adjustment for treatment arms (IMN and IFA), age of patients, and *CCR5^+^*, *HLA-B*^∗^*27^+^/HLA-B*^∗^*57^+^* genotypes affected on the Cox model analysis marginally.

The immunovirological response to the antiretroviral therapy was not significantly different between carriers and noncarriers of the allele *−35 C* in each measure of the surrogate markers of disease progression (CD4^+^ T lymphocyte counts and RNA HIV-1 levels measured every three months during 36 months of follow-up). The SNP did not predict CD4^+^ T cell recovery after 36 months on ART.

Finally, although HIV-1 DNA levels, measured retrospectively at the baseline pre-ART and one month post-ART were significantly different between patients with and without the protective allele, the measurements of HIV-1 DNA levels at the end of the study were not significantly different (*p* = 0.458).

## 4. Discussion

HIV-1 infection exhibits a considerably phenotypic heterogeneity that may be attributed to a complex interplay between viral, environmental, and host genetic factors. Host genes have been extensively explored by several genome-wide association studies, which compiled common human genetic variations across diverse continental populations. More than 16 GWAS targeting various HIV-linked phenotypes have been published since 2007 [1–5, 20]. Surprisingly, only the two HIV-1 chemokine coreceptors and *HLA loci* have exhibited consistent and reproducible statistically significant genetic associations. GWAS focusing on viral load control allowed to identify the association between plasma RNA viral load and the polymorphism *rs9264942* located 35 kb upstream *HLA-C*. Potential association of this SNP with HIV-1 reservoirs, particularly HIV-1 DNA levels had not been investigated. HIV-RNA level from plasma and HIV-1 DNA level from PBMCs are closely related variables but each one provides slightly different information about the replicative history and spread of the virus [21–23]. Both viral markers do not necessarily show the same trend.

**Figure 2:**
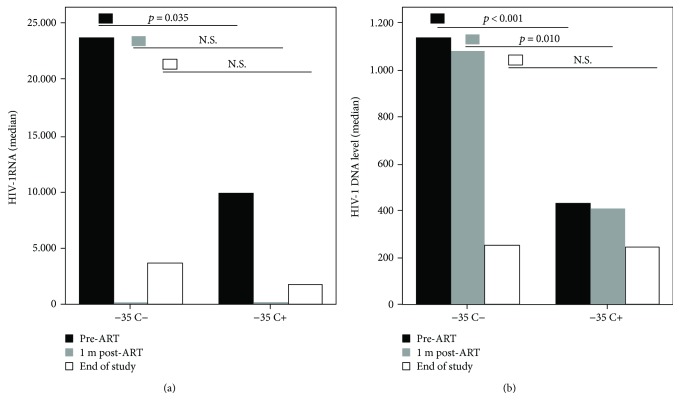
Graph showing the median of HIV-RNA viral load (a) and the median of HIV-1 DNA reservoirs (b) over time (pre-ART, one month post-ART, and at the end of the study) in the patients carrying the protective allele −35 C+ (genotypes CC + CT) and patients noncarrying −35 C (genotype TT); *p* values are indicated.

Here, we have retrospectively genotyped the −*35 C/T* polymorphism *rs9264942* in 183 HIV-1 chronically infected asymptomatic subjects and have confirmed the association of the −*35 C/T* variant with the HIV-1 viral control. In our untreated patients, the protective allele −*35 C* is associated with lower viremias as previously demonstrated and also with lower levels of cellular reservoirs of HIV-1 DNA. Even one month posttherapy, when the majority (78.5%) of the 183 patients in our cohort showed complete suppression of HIV-1 RNA viremia, they maintained significantly different amounts of HIV DNA load between carriers and noncarriers of the protective allele *−35 C*. However, the trend for diminished HIV-1 DNA levels did not reach statistically significance at the end of the study STIR-2102, in response to antiviral therapy after thirty-six months on therapy. Carriers and noncarriers of the allele −*35 C* reached the study endpoint without showing statistically significant differences. Probably the allele −*35 C* has a moderate impact on the viral control only in the natural history of the infection, disappearing when treated with antiretrovirals (Figure 2). The mechanisms that regulate HLA-C expression and the link between this molecule and HIV infection are still not fully understood. Higher levels of HLA-C associated to the protective allele *−35 C* may be important for the viral control in natural history of infection, involving both lower viremias and lower cellular HIV-1 reservoirs, possibly promoting effective CTL recognition and lysis of HIV-infected cells and modulating NK cell activity through the interaction with KIR receptors [24]. Whether HLA-C expression levels are directly responsible for the protective effect of −35 SNP (or 263 insertion/deletion polymorphism) or the strong linking disequilibrium between the protective variant −*35 C* and other protective genes in the *HLA* locus exerts viral control is still unclear. This is extremely difficult to unravel; therefore, we cannot exclude the possibility that some protective *HLA*-alleles have an effect on the viral control independently or in conjunction with −35 SNP [20, 25]. Certain *HLA* variants have been previously associated with viral control such as the protective alleles *HLA-B*^∗^*57* and *HLA-B*^∗^*27* [1–4]. In our study, we have also investigated these alleles (data not shown): eight patients carried the allele *HLA-B*^∗^*57* and other 8 patients carried the allele *HLA-B*^∗^*27.* We did not find any protective effect of these alleles on HIV-1 RNA or HIV-1 DNA viral load before or after the antiretroviral treatment. Probably, the statistical power of our study was limited taking into account the low frequency of both *HLA-B*^∗^*57* and *HLA-B*^∗^*27* in our population and the sample size. Therefore, our data cannot demonstrate an additive and independent protective effect of the alleles *HLA-B*^∗^*57*, *HLA-B*^∗^*27*, and −*35 HLA-C*.

Finally, synergistic interactions between loci may affect outcome after infection, as suggested by associations of specific, functionally relevant HLA and KIR variants with HIV disease outcomes and these require further consideration as well. Several association studies have shown that certain HLA/KIR pairs of genotypes (*HLA-Bw4/KIR3DL* and *HLA-Bw4/KIR3DS1*) are associated with lower rates of disease progression [26–28]. As HLA-C is the natural ligand for the inhibitory receptors KIR2DL1 and KIR2DL2/3, it would be worthy to study HLA-C group haplotypes (C1/C2) in relation with the corresponding polymorphic receptors KIR, in addition to the polymorphism *rs9264942*. Inhibitory signals derived from the KIR/HLA interactions play a pivotal role in discriminating normal from pathologic tissue and NK cell responses resulting in target infected-cell lysis [29]. To date, it is not clear the relevance of HLA-C levels and haplotypes in combination with different KIR in the HIV viral control.

The design of future studies might consider that the host and viral genetic variation are operating in an interacting system and probably each factor is not in itself sufficient to confer perdurable or complete protection for the disease outcome [30]. Considering the dynamic nature of host-pathogen interactions can shape optimized approaches to long-term HIV-1 management.

## 5. Conclusions

HIV-1 RNA viral load and HIV-1 DNA are both, in our study, variables significantly different between carriers and noncarriers of the protective allele −35 C before ART. Higher levels of HLA-C associated to the protective allele −35 C may be important for the viral control in natural history of infection, involving both lower viremias and lower cellular HIV-1 reservoirs, possibly promoting effective CTL recognition and lysis of HIV-infected cells and modulating NK cell activity through the interaction with KIR receptors. This protective effect of the −35 C allele on viral control was not maintained after the antiretroviral treatment. Probably, this allele has a moderate impact on the viral control only in the natural history of the infection, disappearing when treated with antiretrovirals (Figure 2).

## Figures and Tables

**Table 1:** Baseline characteristics for the cohort of patients (study STIR-2102).

	ART + HIV-immunogen	ART + placebo	Overall
Number of subjects	86	97	183
Age, mean (S.D.) (years)	35 (8)	33 (7)	34 (7)
Gender, *N* (%)			
Male	61 (70.9)	65 (67.0)	126
Female	25 (30.0)	32 (33.0)	57
CD4^+^ T cells, mean (S.D.), cells × 10^−6^ L^−1^	405.1 (72.1)	401.0 (74.9)	402.9 (73.4)
HIV-1 RNA, median (range), copies per mL	14,150 (459,801)	14,650 (243,801)	14,525 (459,801)

S.D.: standard deviation.

**Table 2:** Frequencies of the genotypes for the SNP rs9264942 in the *HLA-C* 5′ region, where T is the major allele and C is the minor allele. HIV-1 RNA and HIV-1 DNA levels are shown in the patients naïve for antiretroviral therapy (pre-ART), one month (1 m) post-ART, and at the end of the study (follow-up: 36 months).

rs9264942 genotypes		Frequency *N* (%)	HIV-1 RNA, median (min–max)			HIV-1 DNA, median (min–max)		
			Pre-ART	1 m post-ART	End of study	Pre-ART	1 m post-ART	End of study
Total *N* = 183	TT	54 (29.5%)	24,976 (200–460,000)	200 (200–60,000)	5400 (200–56,543)	927.5 (250–32,560)	1080 (250–10,645)	250 (250–2570)
	CT	95 (51.9%)	11,450 (200–274,500)	200 (200–25,000)	1125 (200–46,200)	520.0 (250–12,985)	440 (250–18,855)	250 (250–10,120)
	CC	34 (18.6%)	9250 (200–206,500)	200 (200–16,100)	5252 (200–41,200)	448.5 (250–17,425)	420 (250–7475)	250 (250–930)
			N.S. *p* = 0.068	N.S.	N.S.	**p** = 0.001	**p** = 0.020	N.S.

Median and minimum–maximum values of viremia and cellular-associated viral loads are indicated for the respective genotypes, HIV-1 RNA in copies/mL, and HIV-1 DNA in copies/10^6^ PBMCs. The *p* values are for median comparisons; N.S.: not significant.

**Table 3:** Frequencies of the individuals carrying the protective allele rs9264942–35 C (genotypes CC + CT) versus noncarriers of this allele (genotype TT). HIV-1 RNA and HIV-1 DNA levels are shown in the patients naïve for antiretroviral therapy (pre-ART), one month (1 m) post-ART, and at the end of the study (follow-up: 36 months).

rs9264942 (T > C) −35 C allele (genotypes)		Frequency (%)	HIV-1 RNA median (min–max)			HIV-1 DNA median (min–max)		
			Pre-ART	1 m post-ART	End of study	Pre-ART	1 m post-ART	End of study
Total *N* = 183	Noncarriers (TT)	54 (29.5%)	24,976 (200–460,000)	200 (200–60,000)	3600 (200–56,543)	927,5 (250–32,560)	1080 (250–10,645)	250 (250–2570)
	Carriers (CT + CC)	129 (70.5%)	10,100 (200–274,500)	200 (200–25,000)	1730 (200–132,000)	500 (250–17,425)	425 (250–18,855)	250 (250–15,920)
			**p** = 0.035	N.S.	N.S.	**p** < 0.001	**p** = 0.010	N.S.

Median and minimum–maximum values of viremia and cellular-associated viral loads are indicated for the respective genotypes, HIV-1 RNA in copies/mL, and HIV-1 DNA in copies/10^6^ PBMCs. The *p* values are for median comparisons; N.S.: not significant.

**Table 4:** Cox regression model for the virological failure during antiviral therapy.

Variable	HR (95% CI)	Endpoint *p* (%)	*p*
HIV-1 RNA (log_10_ copies/mL)^a^	1.86 (1.27–2.72)	65.5	0.001
HIV-1 DNA (log_10_ copies/10^6^ PBMC)^a^	1.74 (1.10–2.77)	63.0	0.018
Protective allele −35 C^b^	0.90 (0.54–1.50)	47.3	0.698

Cox regression model for the virological failure according to the presence of the protective allele −35 C for the SNP rs9264942 adjusting for HIV-1 RNA levels and HIV-1 DNA levels. Hazard ratio (HR), 95% confidence intervals (CIs), endpoint *p* (probability to reach an endpoint, as defined by HR/(1 + HR)), and *p* values are shown. ^a^HRs calculated for a 1-log_10_ increased. ^b^HR calculated for the allele −35 C as a categorical variable (presence of the allele −35 C for the SNP rs9264942[T > C]).

## Additional Points

*Highlights*. (1) The polymorphism *rs9264942* (C/T) impacts on HIV-1 DNA reservoirs. (2) The *rs9264942* allele C is associated with lower levels of HIV-1 DNA. (3) This protective effect is observed in untreated patients. (4) The SNP *rs9264942* is not associated with the viral control after 36 months on ART. (5) The SNP *rs9264942* do not influence T cell recovery in response to ART
